# Impact of genetic markers related to hyper-HDL cholesterol on the prevalence of myocardial infarction: a KoGES study

**DOI:** 10.1016/j.jlr.2025.100777

**Published:** 2025-03-13

**Authors:** Sung-Bum Lee, Kyung-Won Hong, Byoungjin Park, Dong-Hyuk Jung

**Affiliations:** 1 Department of Family Medicine, Soonchunhyang University Bucheon Hospital, Bucheon, Republic of Korea; 2 Institute of Advanced Technology, THERAGEN HEALTH Co., Ltd., Seongnam-si, Gyeonggi-do, Republic of Korea; 3 Department of Family Medicine, Yongin Severance Hospital, Yongin-si, Republic of Korea

**Keywords:** myocardial infarction, hyper-HDL cholesterol, SNP, GWAS, KoGES

## Abstract

Recent studies have shown that hyper-high-density lipoprotein cholesterol (HDL-C) is associated with cardiovascular disease risk and all-cause mortality, a phenomenon known as the HDL-C paradox. Several genes have been reported to show relationships between increased HDL-C and myocardial infarction (MI) risk. We investigated the genetic predisposition of lipid metabolism influencing MI. The study dataset was from the Korean Genome and Epidemiology cohort obtained from the National Biobank of Korea, with an initial population of 68,806 individuals. We categorized samples based on HDL-C levels into hypo-HDL-C (n = 25,884), normal-HDL-C (n = 41,117), and hyper-HDL-C groups (n = 1,805). We conducted genome-wide association studies for each group and the total sample. Significant associations were defined using genome-wide significant level and suggestive level. The lead SNP of each locus was selected for further interpretation. This analysis included 2,014 (2.6%) MI patients. Using multivariable logistic regression, we evaluated the association of 7,877 SNPs in nine loci. We identified six SNPs significantly related to both hypo- and hyper-HDL groups, one SNP associated with hyper-HDL, and six SNPs associated with hypo-HDL group. Additionally, we found three SNPs associated with MI prevalence in the hyper-HDL group, including one significant SNP and two suggestive SNPs. Contrary to the traditional view of HDL-C as protective, this study identified genetic variants that increase MI risk by more than six-fold. These SNPs could play a role as important markers for detecting MI in hyper-HDL cholesterol group.

High-density lipoprotein cholesterol (HDL-C), often referred to as 'good cholesterol,' has traditionally been considered a protective factor against cardiovascular disease (CVD), including myocardial infarction (MI) (1). However, recent studies have challenged this conventional understanding, suggesting that exceptionally hyper-HDL-C levels may not always confer cardiovascular benefits and, in some cases, may even be associated with adverse outcomes (2). Study examining hyper-HDL-C, characterized by abnormally high levels of HDL-C, has provided conflicting evidence regarding its impact on MI risk (3).

Molecular analyses have identified variants in cholesteryl ester transfer protein (*CETP*) and apolipoprotein AI genes correlating with HDL-C concentrations (4), while ATP-binding cassette transporter A1 (*ABCA1*) variants influence HDL particle heterogeneity (5). These genetic determinants modify both HDL-C quantities and atheroprotective functions, with recent evidence indicating posttranslational modifications that may alter vasoprotective properties (6). Despite its clinical relevance, the molecular and physiological mechanisms linking hyper-HDL-C to MI remain poorly understood.

In this study, we aim to investigate the genetic determinants of hyper-HDL-C and their potential implications for MI. Utilizing data from a large-scale Korean cross-sectional cohort [Korean Genome and Epidemiology Study (KoGES)], we performed a genome-wide association study (GWAS) to identify SNPs associated with hyper-HDL-C. Subsequently, we conducted sequential analyses to examine the effects of these identified genetic variants on MI. This research seeks to provide novel insights into the genetic basis of hyper-HDL-C and its role in cardiovascular pathology, particularly within the context of the Korean population.

## Materials and Methods

### Study population

The study participants were drawn from the KoGES cohort, whose detailed cohort profiles previously reported (7). Through the Korean Biobank Resource, we obtained genomic and clinical epidemiological data from 72,299 participants. We excluded samples with missing values for HDL-C levels or MI diagnosis (n = 3,083) and those lacking key covariates, such as body mass index (BMI), smoking status, alcohol consumption, and regular exercise (n = 410). The final dataset comprised 68,806 participants for subsequent analyses. This study was approved by the institutional review board of Yong-In Severance Hospital (IRB No: 2020-0040-002). This investigation was performed based on the Declaration of Helsinki.

### Data collection

Trained technicians performed anthropometric measurements, and interviewers collected lifestyle data through questionnaires. BMI was calculated as weight (kg) divided by height squared (m^2^). Waist circumference was measured at the midpoint between the lowest rib and the iliac crest. Blood samples were collected after a minimum 8-h fast. We estimated alcohol consumption (g/day) based on the frequency and type of alcoholic beverages consumed. Smoking status was categorized as nonsmoker, past smoker, or current smoker. Regular exercise was defined as performing at least 30 min of physical activity daily. Trained technicians measured blood pressure using a mercury sphygmomanometer after participants rested for at least 5 min in a sitting position. Systolic blood pressure, diastolic blood pressure, and pulse rate were measured twice on both arms and averaged (8).

Hypertension was defined as systolic blood pressure ≥ 140 mmHg, diastolic blood pressure ≥ 90 mmHg, or a prior diagnosis (9). Diabetes mellitus was defined as fasting glucose ≥ 126 mg/dl, HbA1c ≥ 6.5%, or a prior diagnosis. Dyslipidemia was defined as meeting any of the following criteria: total cholesterol ≥ 200 mg/dl, triglycerides (TGs) ≥ 150 mg/dl, low-density lipoprotein cholesterol (LDL-C) ≥ 130 mg/dl, HDL-C < 40 mg/dl for men or < 50 mg/dl for women, or a prior diagnosis. MI was defined based on self-reported history of diagnosis or treatment (10). Patients with CVD was defined as those who answered “yes” to histories of MI or ischemic stroke (10).

### Genotype analysis

Genotype data were obtained from the Center for Genome Science of the Korea National Institute of Health using the Korea Biobank Array (KORV 1.0, Affymetrix, Santa Clara, CA) (11). Quality control procedures included filtering by call rate (>97%), minor allele frequency (MAF > 1%), and Hardy–Weinberg equilibrium *P* value > 1 × 10^-5^ (12). Postquality control, genotypes were phased using ShapeIT v2, and imputation was performed using IMPUTE v2 with the 1,000 Genomes Phase 3 reference panel. Variants with imputation quality scores < 0.4 or MAF < 1% were excluded, leaving 7,975,321 SNPs across chromosomes 1–22 for GWAS.

### Statistical analysis

Participants were categorized into HDL-C groups as follows:

Men: hyper-HDL cholesterolemia: HDL > 68 mg/dl; hypo-HDL cholesterolemia: HDL < 43 mg/dl; normal-HDL-C: HDL 43–68 mg/dl.

Women: hyper-HDL cholesterolemia: HDL > 97 mg/dl; hypo-HDL cholesterolemia: HDL < 51 mg/dl; normal-HDL-C: HDL 51–97 mg/dl.

To identify genetic markers associated with HDL-C extremes, GWAS was performed for normal versus hyper-HDL and normal versus hypo-HDL groups. Subsequent GWAS analyses were conducted for MI within each HDL group (hyper-HDL, normal, hypo-HDL). Genome-wide significance was set at *P* < 5 × 10^−8^. Logistic regression was used under an additive genetic model, with adjustments for covariates including sex, age, BMI, smoking status, alcohol consumption, regular exercise, and the first two principal components (PC1, PC2) to account for population structure using the genome-wide principal component analysis (12). All statistical analyses were conducted using PLINK software (v1.9).

## Results

### Baseline characteristics of the study population

From the initial 72,299 individuals, we excluded participants with missing values for HDL-C, BMI, and those who did not complete questionnaires about smoking, drinking, physical activity, and MI history. The final study population comprised 68,806 participants, who were divided into three groups based on HDL levels: hypo-HDL (n = 25,884), normal-HDL (n = 41,117), and hyper-HDL (n = 1,805). The prevalence of MI was 3.7% (968 participants) in the hypo-HDL group, 2.4% (1,003 participants) in the normal-HDL group, and 2.4% (43 participants) in the hyper-HDL group (Table 1).

**Table 1:** Characteristics of population

Variables	Total Population	Hypo-HDL Group	Normal-HDL Group	Hyper-HDL Group	*P* Value
N	68,806	25,884	41,117	1,805	
Age (years)	54.1 ± 8.3	55.0 ± 8.4	53.5 ± 8.2	55.4 ± 8.6	<0.001
Female (%)	44,191 (64.2)	17,616 (68.1)	26,342 (38.3)	233 (12.9)	<0.001
Myocardial infarction (n, %)	2014 (2.9)	968 (3.7)	1,003 (2.4)	43 (2.4)	<0.001
Cardiovascular disease (n, %)	2,844 (4.1)	1,342 (5.2)	1,436 (3.5)	66 (3.7)	<0.001
Hypertension (n, %)	14,011 (20.4)	6,289 (24.3)	7,380 (18.0)	342 (19.0)	<0.001
Type 2 diabetes (n, %)	4,796 (7.0)	2,369 (9.2)	2,313 (5.6)	114 (6.3)	<0.001
Dyslipidemia (n, %)	6,777 (9.9)	2,650 (10.2)	3,970 (9.7)	157 (8.7)	0.001
Lifestyle					
Drinking status (n, %):					<0.001
Never/	35,316 (51.33)/	15,225 (58.82)/	19,812 (48.18)/	279 (15.46)/	
Quit/	2880 (4.19)/	1,279 (4.94)/	1,519 (3.69)/	82 (4.54)/	
Current	30,610 (44.49)	9,380 (36.24)	19,786 (48.12)	1,444 (80.0)	
Smoking status (n, %):					<0.001
Never/	49,528 (71.98)/	19,191 (74.14)/	29,678 (72.18)/	659 (36.51)/	
Quit/	10,976 (15.95)/	3,527 (13.63)/	6,709 (16.32)/	740 (41.0)/	
Current	8,302 (12.07)	3,166 (12.23)	4,730 (11.5)	406 (22.49)	
Exercise status (n, %):					<0.001
No/	33,627 (48.9)/	13,646 (52.7)/	19,270 (46.9)/	711 (39.4)/	
Yes	35,179 (51.1)	12,238 (47.3)	21,847 (53.1)	1,094 (60.6)	
Anthropometric traits					
Body mass index (kg/m^2^)	24.0 ± 2.9	24.7 ± 2.9	23.6 ± 2.9	22.6 ± 2.7	<0.001
Waist circumference (cm)	81.2 ± 8.7	83.1 ± 8.4	80.1 ± 8.7	79.7 ± 8.0	<0.001
Systolic blood pressure (mmHg)	122.1 ± 15.3	122.8 ± 15.5	121.6 ± 15.2	124.2 ± 15.0	<0.001
Diastolic blood pressure (mmHg)	75.9 ± 10.0	76.2 ± 10.0	75.6 ± 10.0	77.4 ± 9.8	<0.001
Biochemical traits					
Fasting plasma glucose (mg/dl)	95.2 ± 20.0	96.8 ± 21.9	94.1 ± 18.6	97.0 ± 20.5	<0.001
HbA1c (%)	5.7 ± 0.8	5.9 ± 0.9	5.7 ± 0.7	5.6 ± 0.7	<0.001
hs-CRP (mg/dl)	0.26 ± 1.28	0.38 ± 1.74	0.19 ± 0.09	0.19 ± 0.74	<0.001
Fasting insulin (μU/ml)	7.61 ± 4.59	8.12 ± 4.83	7.18 ± 4.34	5.98 ± 2.89	<0.001
Total cholesterol (mg/dl)	197.5 ± 35.7	190.9 ± 35.9	201.1 ± 34.9	209.0 ± 35.2	<0.001
HDL cholesterol (mg/dl)	52.8 ± 13.1	41.0 ± 5.7	59.0 ± 10.1	80.8 ± 12.4	<0.001
LDL cholesterol (mg/dl)	119.6 ± 32.1	118.5 ± 32.5	120.8 ± 31.8	110.8 ± 32.0	<0.001
LDL/HDL ratio	2.38 ± 0.84	2.91 ± 0.85	2.10 ± 0.65	1.40 ± 0.45	<0.001
Triglyceride (mg/dl)	129.0 ± 88.8	165.4 ± 109.8	107.0 ± 64.3	88.7 ± 52.1	<0.001
r-glutamyltransferase (U/L)	30.7 ± 41.2	29.4 ± 33.8	30.5 ± 42.5	53.6 ± 80.6	<0.001
AST (U/L)	24.1 ± 21.6	24.3 ± 30.4	23.8 ± 13.3	28.0 ± 23.4	<0.001
ALT (U/L)	22.7 ± 22.6	24.1 ± 27.4	21.8 ± 17.6	24.5 ± 39.6	<0.001
ALP (U/L)	163.2 ± 105.6	162.9 ± 109.1	163.9 ± 96.0	154.0 ± 206.1	<0.001
Albumin (g/dl)	4.6 ± 0.5	4.5 ± 0.6	4.6 ± 0.4	4.7 ± 0.3	<0.001
BUN (mg/dl)	14.6 ± 4.1	14.4 ± 4.3	14.6 ± 3.9	15.5 ± 4.2	<0.001
Creatinine (mg/dl)	0.82 ± 0.21	0.83 ± 0.23	0.81 ± 0.2	0.91 ± 0.27	<0.001
Uric Acid (mg/dl)	4.7 ± 1.3	4.8 ± 1.3	4.6 ± 1.3	5.2 ± 1.3	<0.001

ALT, alanine transaminase; AST, aspartate transaminase.

Table 1 presents the characteristics of the study population. The mean age (± standard deviation) was 54.1 ± 8.3 years in the total population, with 55.0 ± 8.4 years in the hypo-HDL group, 53.5 ± 8.2 years in the normal-HDL group, and 55.4 ± 8.6 years in the hyper-HDL group (*P* value < 0.001). The hyper-HDL group showed significantly lower values for dyslipidemia prevalence, BMI, waist circumference, HbA1c, fasting insulin, LDL-C, LDL/HDL ratio, and TGs compared to other groups. Conversely, this group had significantly higher values for total cholesterol, HDL-C, r-GT, albumin, and BUN compared to the other groups.

The prevalence of MI was the highest in the hypo-HDL group compared to both normal-HDL and hyper-HDL groups. To investigate the potential genetic factors underlying this relationship between baseline HDL-C concentration and MI, we conducted GWAS.

### Genome-wide association studies for hypo- and hyper-HDL-C

The primary aim of this study was to identify genetic markers associated with increased MI risk in individuals with hyper-HDL-C. As a preliminary step, we conducted GWAS to identify genetic markers significantly associated with both hypo- and hyper-HDL-C. A total of 7,877 SNPs in nine loci were evaluated in order to find the genetic association of dyslipidemia with MI (supplemental Table S1). We compared individuals in the hypo- and hyper-HDL groups to those in the normal-HDL group, which served as our control.

**Fig. 1:**
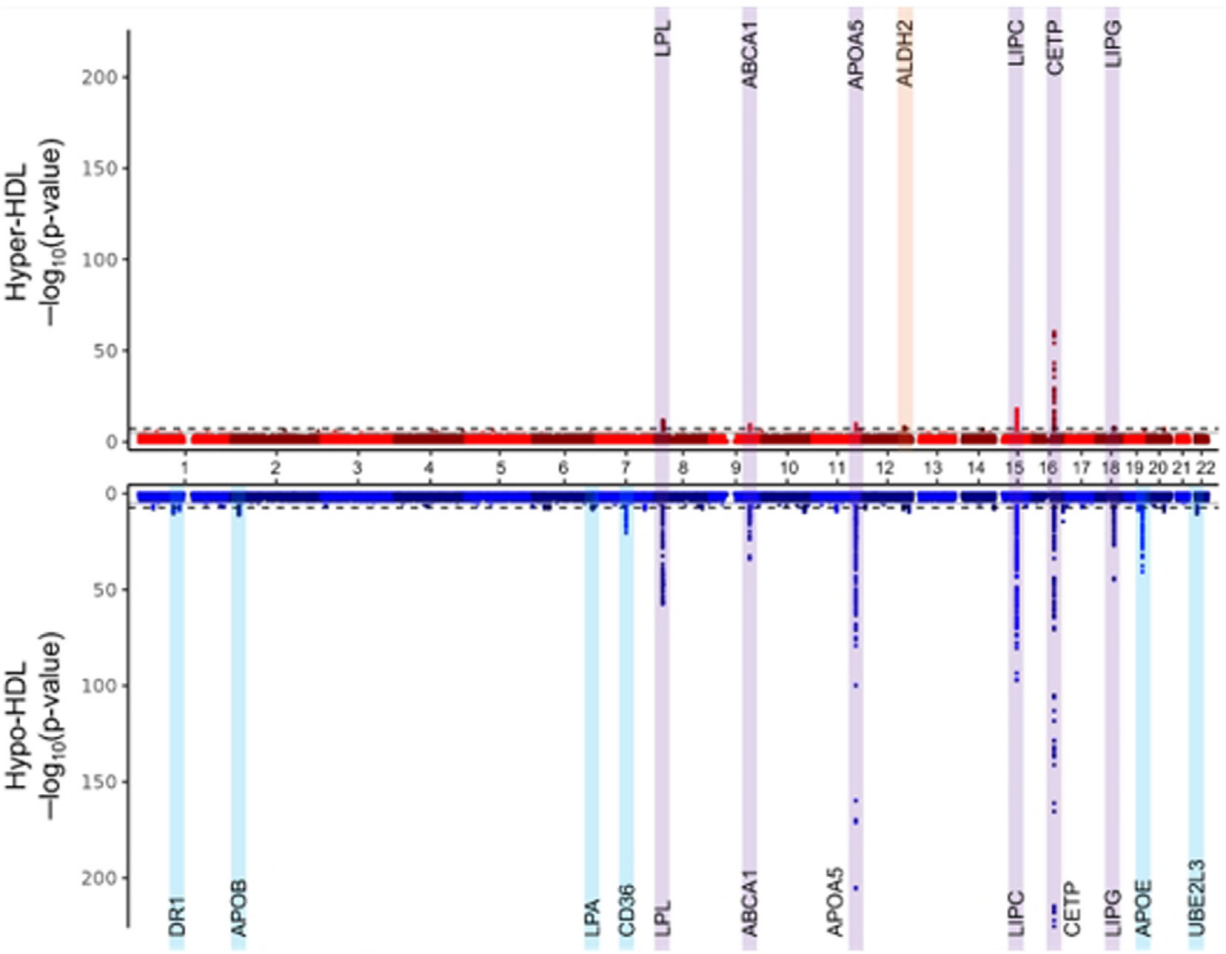
Miami plot for the genome-wide association study results. The GWAS results for the normal-HDL cholesterol group versus hyper-HDL cholesterol group are represented in the upper red plot, while the hypo-HDL cholesterol group versus the normal-HDL cholesterol group results are depicted in the lower blue plot. Each dot in the Miami plot represents the -log10 transformed *P* value from the genome-wide logistic regression analysis. Genomic loci passed the genome-wide significance threshold (5 × 10^−8^) are highlighted with colored boxes and annotated with their candidate genes. The colors of the boxes indicate the significance across groups: Purple boxes represent loci that are significant in both the hyper and hypo groups; orange boxes indicate loci significant only in the hyper group; sky blue boxes denote loci significant only in the hypo group. The association statistics for the lead SNPs in each locus are detailed in Table 2.

The GWAS identified 12 loci significantly associated with hypo-HDL cholesterolemia and seven loci significantly associated with hyper-HDL cholesterolemia, as illustrated in the Miami plots in Fig. 1. We performed in silico analyses for each significant locus to interpret how nearby candidate genes might influence HDL-C levels (Table 2). None of the identified SNPs showed significant association with MI risk in the hyper-HDL group. Therefore, we conducted separate GWAS to identify genetic markers influencing MI risk in each of the hypo-, normal-, and hyper-HDL groups.

**Table 2:** Lead SNPs identified through GWAS as showing significant associations in either the hypo-HDL cholesterol group or the hyper-HDL cholesterol group compared to the normal-HDL cholesterol group

SNP ID	Chr		BP	Minor Allele	1000G-Based MAF			Nearby Gene	Normal-HDL Versus Hypo-HDL		Normal-HDL Versus Hyper-HDL	
					Asian	European	American		OR (95% CI)	*P* Value	OR (95% CI)	*P* Value
Significantly associated with both groups												
rs17482753	8		19832646	T	0.12	0.13	0.06	LPL	0.75 (0.72–0.77)	3.33 × 10^−58^	1.37 (1.25–1.51)	1.95 × 10^−11^
rs1883025	9		107664301	T	0.24	0.24	0.27	ABCA1	1.18 (1.15–1.21)	8.15 × 10^−35^	0.76 (0.70–0.83)	4.46 × 10^−10^
rs651821	11		116662579	T	0.71	0.92	0.85	APOA5	1.47 (1.44–1.51)	2.53 × 10^−206^	0.76 (0.70–0.83)	1.83 × 10^−10^
rs1077835	15		58723426	G	0.42	0.21	0.43	LIPC	0.78 (0.76–0.80)	7.01 × 10^−98^	1.37 (1.28–1.47)	1.36 × 10^−18^
rs17231,506	16		56994528	T	0.18	0.29	0.29	CETP	0.59 (0.57–0.61)	4.36 × 10^−226^	1.94 (1.79–2.10)	1.87 × 10^−59^
rs9953437	18		47120600	A	0.43	0.05	0.16	LIPG	0.85 (0.83–0.87)	1.29 × 10^−45^	1.23 (1.14–1.32)	8.81 × 10^−9^
Significantly associated with hyper-HDL cholesterolemia												
rs671	12		112241766	A	0.17	0.00	0.00	ALDH2	1.04 (1.01–1.08)	9.11 × 10^−3^	0.70 (0.62–0.79)	5.61 × 10^−9^
Significantly associated with hypo-HDL cholesterolemia												
rs4847240	1	93817946		A	0.36	0.36	0.42	DR1	0.93 (0.90–0.95)	5.57 × 10^−11^	1.04 (0.97–1.12)	2.39 × 10^−1^
rs36145916	2	21179378		T	0.15	0.44	0.52	APOB	1.12 (1.08–1.15)	7.69 × 10^−12^	0.95 (0.86–1.05)	3.49 × 10^−1^
rs9355291	6	160989288		T	0.50	0.86	0.61	LPA	1.07 (1.05–1.10)	4.28 × 10^−9^	0.94 (0.88–1.01)	9.52 × 10^−2^
rs75326924	7	80286003		T	0.01	0.00	0.00	CD36	0.80 (0.77–0.84)	4.42 × 10^−21^	1.18 (1.03–1.34)	1.35 × 10^−2^
rs429358	19	4,5411941		C	0.09	0.16	0.10	APOE	1.30 (1.25–1.35)	2.43 × 10^−41^	0.76 (0.66–0.87)	5.31 × 10^−5^
rs4821130	22	21980894		T	0.51	0.19	0.38	UBE2L3	1.08 (1.06–1.11)	3.44 × 10^−11^	0.97 (0.90–1.04)	3.95 × 10^−1^

### Genetic relationship between HDL-C levels and MI

**Fig. 2:**
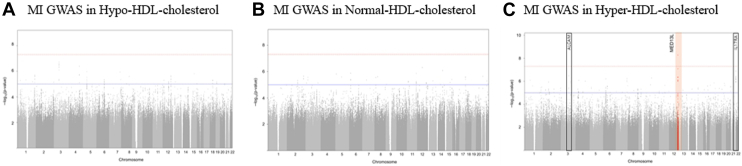
Manhattan plot for myocardial infarction case-control genome-wide association studies across three HDL cholesterol groups. A: Hypo-HDL cholesterol group; (B) normal-HDL cholesterol group; (C) hyper-HDL cholesterol group. The Manhattan plots illustrate the -log10 transformed *P* values from the GWAS results for each HDL cholesterol group. The x-axis represents the chromosome number where each genetic variant is located. The red horizontal line denotes the genome-wide significance threshold (*P* value = 5 × 10^−8^), while the blue horizontal line represents the genome-wide suggestive threshold (*P* value = 1 × 10^−5^). Genomic loci that pass the genome-wide significance threshold are highlighted with red boxes and annotated with the corresponding gene names. Detailed information about the lead SNPs for these significant loci can be found in Table 3.

We performed GWAS for MI in each of the hypo-, normal-, and hyper-HDL-C groups. The results of these analyses are depicted as Manhattan plots in Fig. 2A–C. Using the genome-wide significance threshold (*P* value < 5 × 10^−8^), no significant SNPs were identified in the hypo- or normal-HDL-C groups. However, in the hyper-HDL-C group, we identified rs74457740, a SNP located in the *MED13L* gene region, which was significantly associated with an increased risk of MI (odds ratio: 6.064, 95% CI: 3.31–11.11, *P* value = 5.23 × 10^−9^, Table 3). Furthermore, two suggestive SNPs were also associated with MI in the hyper-HDL-C group and positioned in 3q13.11 region (odds ratio: 8.263 95% CI: 3.673–18.590 *P* value = 3.295 × 10^−7^) and 22q11.1 region (odds ratio: 5.745, 95% CI: 3.000–11.000, *P* value = 1.330 × 10^−7^).

**Table 3:** Lead SNPs showing significant association with MI in the hyper-HDL cholesterol group

SNP ID	Chr	BP	Minor Allele	Asian	European	American	Nearby Gene	Test	OR (95% CI)	*P* Value
rs141602596	3	104444813	A	0.01	0.00	0.00	*ALCAM*	HDL cholesterol level GWAS		
								Normal versus hypo-HDL	0.934 (0.856–1.018)	0.118
								Normal versus hyper-HDL	1.005 (0.768–1.314)	0.972
								MI risk GWAS		
								Total group	1.291 (1.037–1.607)	0.022
								Hypo-HDL group	1.022 (0.716–1.460)	0.905
								Normal-HDL group	1.334 (0.985–1.807)	0.063
								Hyper-HDL group	8.263 (3.673–18.59)	3.295 × 10^−7^
rs74457740	12	116354722	T	0.11	0.08	0.03	*MED13L*	HDL cholesterol level GWAS		
								Normal versus hypo-HDL	0.978 (0.933–1.025)	0.350
								Normal versus hyper-HDL	1.069 (0.929–1.231)	0.351
								MI risk GWAS		
								Total group	1.025 (0.899–1.170)	0.711
								Hypo-HDL group	0.955 (0.784–1.162)	0.645
								Normal HDL group	0.965 (0.798–1.167)	0.712
								Hyper-HDL group	6.064 (3.312–11.11)	5.233 × 10^−9^
rs5748859	22	17567177	A	0.05	0.05	0.04	*IL17RA*	HDL cholesterol level GWAS		
								Normal versus hypo-HDL	0.963 (0.912–1.017)	0.180
								Normal versus hyper-HDL	0.899 (0.756–1.070)	0.231
								MI risk GWAS		
								Total group	1.011 (0.868–1.179)	0.886
								Hypo-HDL group	0.929 (0.738–1.170)	0.532
								Normal HDL group	0.958 (0.769–1.194)	0.705
								Hyper-HDL group	5.745 (3.000–11.000)	1.330 × 10^−7^

**Fig. 3:**
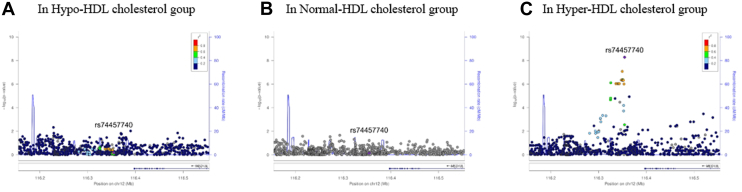
Signal plots for the *MED13L* gene region in the GWAS analysis for myocardial infarction. This figure shows an enlarged view (approximately 1 Mbp) of the *MED13L* gene region, which exhibited the most significant association in the GWAS for myocardial infarction. A: Shows the results for the Hypo-HDL cholesterol group, (B) for the Normal-HDL cholesterol group, and (C) for the Hyper-HDL cholesterol group. Each point on the plot represents a single nucleotide polymorphism (SNP), with the -log10 transformed *P* values of the SNPs plotted on the right y-axis. The color of each point indicates the strength of linkage disequilibrium between the SNP and the lead SNP (rs74457740). The left y-axis represents the recombination rate, which indicates the likelihood of recombination events occurring in this chromosomal region during meiosis. The x-axis shows the genomic location, and the boxes beneath the SNP points represent the functional candidate genes in this region. The direction of mRNA transcription is indicated by arrows, and the exon-intron structures of the genes are visualized with vertical lines (exons) along the horizontal bars representing gene regions.

Upon closer examination of the *MED13L* region in the GWAS results, we observed that the association signal for rs74457740 was specific to the hyper-HDL-C group, forming a distinct cluster of significant signals. This signal was absent in both the hypo- and normal-HDL-C groups (Fig. 3). This finding highlights the unique genetic interplay between the hyper-HDL-C group and MI risk, underscoring the specificity of the identified genetic marker in this context.

### In silico annotation of related genes and functional relevance

We identified seven SNPs that affect HDL-C levels, each previously associated with dyslipidemia. The first SNP, rs17482753, is located near the lipoprotein lipase gene, which is essential for circulating TG hydrolysis, facilitating free fatty acid release and uptake in adipose tissue and muscle (13). The second SNP, rs1883025, is near the *ABCA1* gene, which is crucial for extracellular cholesterol and phospholipid movement, playing a key role in HDL-C formation (14). The third SNP, rs651821, is near the apolipoprotein A5 (*APOA5*) gene, which influences TG metabolism and contributes to lipoprotein lipase activation, with genetic variations significantly affecting TG levels (15). The fourth SNP, rs671, is near the aldehyde dehydrogenase 2 (*ALDH2*) gene, whose polymorphisms significantly influence LDL-C levels in Asian populations, indicating its role in lipid profile regulation (16). The fifth SNP, rs1077835, is near the hepatic lipase (*LIPC*) gene, which encodes an enzyme that facilitates lipoprotein breakdown in the liver and HDL-C remodeling, thereby regulating fatty acid and cholesterol metabolism (17). The sixth SNP, rs17231,506, is located in the *CETP* gene, which plays a crucial role in lipid transport and metabolism. The seventh SNP, rs9953437, is near the endothelial lipase (*LIPG*) gene, which encodes an enzyme involved in TG and phospholipid metabolism in the liver, contributing to fatty acid regulation (18).

Furthermore, we identified three SNPs associated with MI in the hyper-HDL-C group, located in regions 12q24.21, 3q13.11, and 22q11.1, all previously linked to MI risk. The most significant SNP, rs74457740, is located in the 12q24.21 region near its closest functional gene, *MED13L*. This gene has been associated with low heart rate variability (19). The SNP rs141602596 is located in region 3q13.11 near the activated leukocyte cell adhesion molecule (*ALCAM*) gene. *ALCAM* encodes a protein critical for cell adhesion and migration, particularly in T-cell differentiation. While elevated ALCAM levels may enhance excess cholesterol scavenging, its overexpression can induce apoptosis in cardiovascular cells (20). Studies have shown that a 50% increase in ALCAM concentration correlates with a 1.16-fold increase in CVD risk and a 1.45-fold increase in cardiovascular mortality (21). The SNP rs5748859 is located on region 22q11.1 near the interleukin 17A receptor (*IL17RA*) gene. *IL17RA* encodes a type I membrane glycoprotein that binds interleukin (IL) 17A, a proinflammatory cytokine secreted by activated T-lymphocytes (22). Its essential role in inflammatory and autoimmune diseases suggests a potential contribution to MI risk through inflammatory pathways (23).

## DISCUSSION

Dyslipidemia is a disease characterized by abnormalities in blood lipid profiles such as TGs, LDL-C, and HDL-C (24). It commonly presents with elevated TGs or LDL-C and reduced HDL-C leading to complications such as CVD (25). From this perspective, HDL-C has been negatively associated with MI. However, recent study has shown that even excessively high concentration of HDL-C is positively related to MI (26). Therefore, we hypothesized that genetic factors might mediate the relationship between HDL-C and MI risk. Specifically, we proposed that individuals with hyper-HDL-C might have increased MI risk if they carry minor alleles of certain SNPs. Testing this hypothesis, we identified three SNPs associated with significantly higher MI prevalence among participants with elevated HDL-C levels.

The first SNP, rs141602596, is located on 3q13.11, with *ALCAM* as its proximal functional gene. *ALCAM*, a cell adhesion molecule from the immunoglobulin superfamily, is expressed in various cell types, including endothelial and leukocyte cells (27). During inflammation, ALCAM levels increase notably, playing a role in leukocyte transmigration across the CNS endothelium (28). Following acute ischemic stroke, serum ALCAM levels show consistent elevation during the initial days and serve as an independent predictor of outcomes, even after adjusted analysis (29). ALCAM also functions as a surface marker on cardiomyocytes derived from human embryonic stem cells (30). Previous research has demonstrated a significant association between ALCAM levels and cardiovascular death (21). This association may be explained by ALCAM's strong correlation with post-CVD inflammatory markers, including IL-18, NT-proBNP, GDF15, and P-selectin. Additionally, patients with elevated ALCAM levels showed a significantly higher prevalence of heart failure history compared to those with low ALCAM levels, suggesting that elevated ALCAM levels in ACS patients with poor prognoses may be linked to inflammatory response (21).

The second SNP, rs5748859, is located on 22q11.1, with *IL17RA* as its proximal functional gene. IL-17 becomes activated upon binding to the IL-17 receptor site, using a specific signaling pathway to interact with the IL-17RA complex and trigger downstream reactions (31). During infections, IL-17 initiates an inflammatory cascade, leading to the production of several proinflammatory cytokines including IL-6, IL-8, TNF-α, and IL-1β (22). As a highly versatile proinflammatory cytokine, IL-17 plays essential roles in tissue repair, inflammatory disease pathogenesis, cancer progression, and host immunological defenses (32). The IL-17 pathway is particularly crucial in atherosclerosis pathogenesis, a condition closely linked to immune response (33). Studies have shown that IL-17A stimulation in human carotid atherosclerosis results in elevated expression of proinflammatory markers (34). Deletion of IL-17RA can provide protective effects by reducing monocyte adhesion to the aorta wall, a process normally mediated by IL-17A cytokine binding to its receptor. Consequently, when IL-17RA is deleted, emerging atherosclerotic lesions show reduced levels of proinflammatory mediators. In this context, T-cell infiltration into the artery intima, a hallmark of early atherosclerosis, characterizes the initial stages of the disease process (35). The established role of inflammation in atherosclerosis development helps explain why functional variations in genes encoding inflammatory proteins are associated with increased MI risk (36).

The most significant SNP identified was rs74457740, with *MED13L* as its nearest functional gene. A polymorphism with a minor allele in the *MED13L* gene has shown positive association with systolic blood pressure (37). Additional polymorphisms in the *MED13L* gene were identified in mouse strains showing varying susceptibility to autoimmune diseases (38). Furthermore, MED13L is indirectly related to inflammation through its interaction with activating transcription factor 4 (ATF4). The *MED13L* gene functions downstream of *ATF4* and can modulate its expression levels, suggesting involvement in ATF4-regulated pathways. ATF4 participates in inflammatory reactions and regulates the release of inflammatory cytokines, including IL-6, IL-8, and interferon-γ (39). Moreover, GWAS studies have revealed that a region proximal to the *MED13L* gene is associated with type 1 diabetes, a condition closely linked to MI (40). These findings suggest that polymorphisms in *MED13L* may contribute to MI risk through multiple pathways.

HDL-C is commonly known for its protective role against dyslipidemia through two essential mechanisms: inflammation modulation and reverse cholesterol transport (cholesterol efflux). However, a recent study suggests that patients with CVD often have high levels of HDL-C (41), and more recent research indicates a U-shaped association between HDL-C and subclinical atherosclerosis (26). Apolipoprotein A-I, the main component of HDL-C, facilitates cholesterol efflux through interactions with macrophage ATP-binding cassette transporters ABCA1 and ABCG1. However, oxidative stress can damage apolipoprotein A-I, diminishing its cholesterol excretion capacity (42). Furthermore, in an oxidative environment, multiple HDL-C proteins can be modified by oxidative stress, transforming HDL-C from an antiinflammatory to a proinflammatory particle (43). The study demonstrated that HDL-C genetic risk scores accounted for only 6% of HDL-C level variation in the UK Biobank study (43). While this suggests a limited genetic contribution to HDL-C levels overall, hyper-HDL-C can still promote oxidative stress, potentially increasing MI risk in participants carrying the minor alleles of specific SNPs.

Our study has several strengths. First, it was conducted using a large population-based dataset. Additionally, we compared our findings with data from three different ethnic populations (Asian, European, and American) to validate the genetic associations between dyslipidemia and MI. This study is novel in revealing associations between specific SNPs and MI risk in the hyper-HDL-C group, addressing an important gap due to the previous shortage of SNP data in this population. Furthermore, our findings suggest that genetic testing could help identify MI risk among patients with hyper-HDL-C.

Nevertheless, our study has several limitations. First, the cross-sectional nature of our study, as opposed to a cohort design, makes it difficult to establish SNP polymorphisms as predictors of MI. Second, the baseline KoGES study did not include physical activity data, an important factor affecting MI risk. Furthermore, environmental factors such as diet, socioeconomic status, and physical activity can modify epigenetic states and influence gene expression independently of genotype potentially affecting the relationship between genetic variants and MI susceptibility (44).

In conclusion, we identified three SNPs negatively associated with HDL-C levels and four SNPs positively associated with HDL-C levels. We also demonstrated that three SNPs—rs141602596 near the *ALCAM* gene, rs5748859 near the *IL17RA* gene, and rs74457740 near the *MED13L* gene—are genetically associated with MI risk in the hyper-HDL-C group. Further studies are needed to confirm these SNPs' relationship with MI incidence.

## Data availability

The genome study used the dataset originally generated in the KoGES supported by the Korean National Institute of Health (K004EIH). The subject information and SNP genotype data used in this study are owned entirely by the Korea National Institute of Health (KNIH), and disclosure of the raw data to the public without permission is strictly prohibited. In principle, the raw data of subject information and SNP genotype used in this study are available with permission from the Institutional Review Board of KNIH for researchers in Korea who meet confidential data access criteria. These data can also be available for researchers overseas when undertaking an international cooperative research project and when the KNIH approves it.

## Conflicts of interest

The authors declare that they have no known competing financial interests or personal relationships that could have appeared to influence the work reported in this paper.
